# A new bioabsorbable polymer film to prevent peritoneal adhesions validated in a post-surgical animal model

**DOI:** 10.1371/journal.pone.0202285

**Published:** 2018-11-05

**Authors:** Lucie Allègre, Isabelle Le Teuff, Salomé Leprince, Sophie Warembourg, Hubert Taillades, Xavier Garric, Vincent Letouzey, Stephanie Huberlant

**Affiliations:** 1 Department of gynecology and obstetrics, University Hospital of Nîmes, Nîmes, France; 2 Department of Artificial Polymers, Max Mousseron Institute of Biomolecules, CNRS UMR 5247, University of Montpellier 1, Montpellier, France; 3 Surgical and Experimental Department, University of Montpellier, Montpellier, France; Institute of Materials Science, GERMANY

## Abstract

**Background:**

Peritoneal adhesions are a serious surgical postoperative complication. The aim of this study is to investigate, in a rat model, the anti-adhesive effects of a bioabsorbable film of polymer combining polyethylene glycol and polylactic acid.

**Materials and methods:**

Sixty-three animals were randomized into five groups according to the anti-adhesion treatment: Hyalobarrier^®^, Seprafilm^®^, Polymer A (PA), Polymer B (PB), and control. The rats were euthanized on days 5 and 12 to evaluate the extent, severity and degree of adhesions and histopathological changes. Three animals were euthanized at day 2 in PA, PB and control groups to observe the *in vivo* elimination.

**Results:**

Macroscopic adhesion formation was significantly lower in the PA group than in the control group at day 5 (median adhesion score 0±0 vs 9.6 ±0.5 p = 0.002) and at day 12 (0±0 vs 7.3±4 p = 0.02). Furthermore, median adhesion score at day 5 was significantly lower in the PA group than in the Seprafilm group (0±0 vs 4.2± 3.9 p = 0.03). Residence time of PA seems longer than PB.

**Conclusion:**

The PA bioabsorbable film seems efficient in preventing the formation of peritoneal adhesions.

## Introduction

Intraperitoneal adhesions are the most common complication of gynecological surgery, occurring in 50% to 100% of women [1]. They are associated with considerable comorbidity including chronic pelvic pain, dyspareunia, subfertility and bowel obstruction [2]. Adhesions could also be problematic in other specialty as oncologic or pediatric surgery [3]. They have large financial and public health repercussions associated with hospital readmissions costs [4,5] and represent a real public health problem.

Intraperitoneal adhesions are fibrin bands formed following a defective repair of the peritoneum [6]. Following a peritoneal trauma there is an increase in vessel permeability and an exudation of inflammatory cells. This phenomenon leads to the formation of a fibrin matrix between 5 and 7 days which is thereafter replaced by a fibrin band during the second week after surgery [7]. Under normal conditions the fibrin is degraded by activation of fibrinolysis. Peritoneal adhesion is the result of abnormal reduction of the fibrinolysis process and a persistence of the fibrin band is observed [6,8]. Some authors reported that mechanical barrier could decrease adhesion formation by keeping peritoneal surface separate during the first 5–7 days required for re-epithelialization. Adhesion severity is correlated to the type of surgery, but is probably underestimated [9].

Various anti-adhesion devices have been developed under different galenic forms, such as Seprafilm (Sanofi, Paris, France) or Interceed (Johnson & Johnson, New Brunswick, NJ, USA) [10]. Currently, no anti-adhesion agent has shown a superior efficiency compared to the others according to the latest Cochrane library review in terms of adhesion prevention. Furthermore, there is no evidence about their efficiency on chronic pelvic pain, fertility or quality of life [11]. However, efficiency of these agents in preventing incidence and severity of adhesions was established [12] and their use is recommended by the American Society of Reproductive Medicine [13]. In abdominal surgery, a recent meta-analysis showed that the use of oxidized regenerated cellulose and hyaluronate carboxymethylcellulose can safely reduce clinically-relevant consequences of adhesions, but data are scarce about their impact on chronic pelvic pain or fertility [14]. However, the main issue of these barriers is the ease of use and the difficulty of placement especially in laparoscopy or hysteroscopy for the prevention of intrauterine adhesion. Indeed, Seprafilm tend to break and its placement need a learning curve and some application method [15,16]. The gel barriers probably don’t remain on the injuries surfaces and could fail to keep separate these surfaces. Moreover most of gel adhesion barriers are not approved for use in United States [17]. There is a lack of an adhesion barrier easy to use that could be use both in open surgery, laparoscopy and hysteroscopy. Recently, a broad range of polymer have been developed in several medicine domains and represent a technological advance for healthcare [18].

In this work, a bioabsorbable polymer film based on polyethylene glycol (PEG) and poly(D,L-lactide) (PLA) was developed to prevent the formation of post-operative peritoneal adhesions. Anti-adhesion properties of PEG are well-documented, and it is present in several anti-adhesion agents currently on the market [19,20]. PEG is hydrophilic, water soluble, and remains in the human body less than seven days [21]. To prevent rapid degradation and solubilization in biological fluids, it has been chemically combined with PLA, which is hydrophobic, non-water soluble, and biodegradable by hydrolysis of its ester functions. PLA has been used in healthcare for many years, in particular in surgery. [22–24].

Our polymers are triblocks of PLA-PEG-PLA with differing ratios of these two components. Many studies have shown that the residence time of anti-adhesion barriers was a key feature for their efficacy [25–27]. Our *in vitro* results (unpublished data) suggested a variability of degradation time according to the ratio of PLA, as previously demonstrated by Yu et al. in 2011 [28]. The PLA/PEG ratio was varied to obtain a polymer absorbable in 15 days. Characterization of the *in vitro* properties of our polymers will form the subject of another publication.

The anti-adhesion effect of our polymer films was tested on a serosal injury model described in the literature [7]. The main objective of this study is to evaluate anti-adhesion efficiency of our bioabsorbable polymer films and to compare them against other anti-adhesion devices currently on the market.

The secondary objectives were to study the *in vivo* removal of our two polymers and to visualize adhesion formation and peritoneal healing with or without polymer.

## Materials and methods

### Synthesis and characterization of polymers

Polymers films were synthesized by ring-opening polymerization of D,L lactide from Polyethylene Oxide (PEO) 100,000 g.mol-1 as initiator. Typically, 10 g of PEO and various amounts of D,L-lactide (9 and 24 g) were introduced into two flasks, the initial molar ratio of ethyl oxide to lactate units (EO/LA) being respectively 1/1 (Polymer A: PA) and 3/1 (Polymer B: PB). PEO and D,L-lactide were vacuum dried for 24h. Tin (II)-2 ethylhexanoate (0.1% of the number of hydroxyl functions of PEO) were added in dried polymerization flasks. After degassing, the flask were sealed under vacuum and the polymerization was carried out at 130°C for five days. The polymers were recovered by dissolution in dichloromethane and precipitated in cold diethyl ether/ethanol (70/30; v/v). The precipitated polymers were filtered and dried under reduced pressure up to constant weight. The polymer films were shaped by hot pressing with a Carver 4120 press by placing the copolymer in a stainless steel mold heated to 100°C for 10 minutes. A constant pressure of 3 psi was applied for 15 minutes. Polymers films were sterilized with gamma radiation emitted by a cobalt spring (SYNERGY HEALTH, Marseille, France).

Two different polymers of differing PLA chain length with different chemical compositions were tested. PA has longer PLA blocks than PB, rendering it more hydrophobic with a longer elimination time than PB.

Anti-adhesion devices used for comparison in this study were Seprafilm (Sanofi,Paris, France) which is a hyaluronate-carboxymethylcellulose membrane, and Hyalobarrier (Nordic Pharma, France), a hyaluronate gel.

### Animals experimentation

All experiments were conducted in the experimental laboratory of the faculty of medicine of Montpellier. Oncins France Strain A (OFA) albino female rats, weighing 250-300g, aged from 6 to 8 weeks, were purchased from the Charles River Laboratories (L’Arbresle, France). OFA albino rats were chosen because of their robustness and their low cost compared to large animal. Furthermore, many studies have described animal adhesion models in this species [2,6,10].

All investigations were carried out in strict accordance with the recommendations in the Guide for the Care and Use of Laboratory Animals. The protocol was approved by the Committee on the Ethics of the French Ministry of Education and Research (contract number 02367.01, task order 1065). All efforts were made to minimize animal suffering and to use the minimum number of animals necessary to produce reliable scientific data. All the animals were in quarantine for one week prior to treatment. They were housed in individual cages in a room at 22°C with a humidity rate of 55% (+/-10%) with free access to food (SAFE) and tap water. Each cage was marked by an identification number after randomization in the aim to labeled and tracked each animal throughout the experimentation. They were examined, weighed and their litter changed daily, respecting the guide of good practices and animal welfare.

### Surgical procedure

The rats were anesthetized by an intra-peritoneal injection of ketamine (80mg/kg) and xylazine (5mg/kg). They were placed in a dorsal position, the abdominal area was shaved and prepared with iodine solution and then they were draped in a sterile fashion. A subcutaneous injection of xylocaine (0.1%) was made to minimize post-operative pain. An anterior midline incision of 4 cm was made through the abdominal wall and peritoneum, and the abdominal cavity was exposed by metallic retractors. The cecum was exteriorized and abraded with a sterile gauze until an area of 3 cm^2^ was deserosalized, as evidenced by punctuate bleeding without hemostasis. Then, an area of the parietal peritoneum directly anterior to the cecum was excised from the abdominal wall, including a superficial layer of the underlying muscle.

### Insertion of anti-adhesion agents

**Fig 1:**
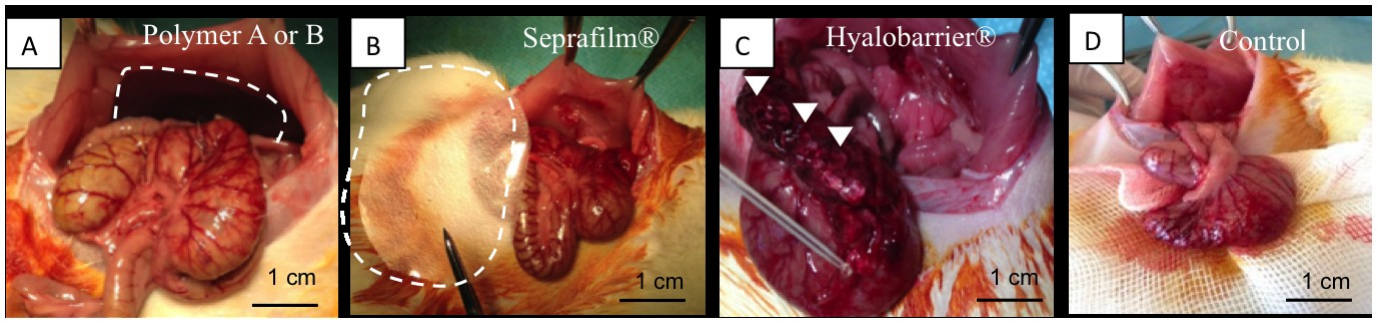
Per operative images showing the positioning of the various anti-adhesion devices. A 4x3.5 cm film of polymer A or B was interposed between the defect and the cecum in group PA and PB (delineated with dashed lines) (A). A 4x3.5 cm film of Seprafilm was interposed in the same way (delineated with dashed lines) (B). One milliliter of Hyalobarrier gel was applied on the abraded cecum (arrow heads) (C). No anti-adhesion agent was used in the control group (D).

The A and B polymer films and Seprafilm were positioned to form a barrier between the damaged peritoneum and the cecum. One ml of Hyalobarrier gel was applied to the surface of the peritoneal defect and the cecum using a catheter and a syringe. In control group, 1 ml of isotonic physiological serum (NaCl 0.9%) was applied to the abdominal cavity. The animals were allocated into five groups via computer-generated randomization: group Hyalobarrier, group Seprafilm, group PA, group PB, group surgery-alone control (Fig 1). The time of euthanasia was also selected by computer-generated randomization.

To avoid any bias in surgery procedure, the surgeon was informed of agent allocation only after cecal abrasion.

Finally, the peritoneal cavity was closed with a continuous suture of 2/0 absorbable suture (Polyglactine 910 suture). The skin was closed with a continuous suture of 3/0 absorbable suture (Polyglactine 910 suture) and the animals were returned to their individual cages. The same type of absorbable suture was used on all animal. Post-operative monitoring was performed daily: assessment of the overall condition, the weight and the intestinal transit, and monitoring of the skin healing. If there were signs that could indicate a physical suffering of the animal (apathy, prostration, weight loss > 15%, evisceration, bowel obstruction), euthanasia was performed. If the animal seemed to suffer during the surgery (reactivity, tachycardia), a second injection of ketamine was performed.

### Macroscopic scoring of peritoneal adhesions

The animals were euthanized at day 5 and day 12 post-operative by an intra-peritoneal injection of pentobarbital (0.5mL/kg). The abdominal cavity was re-opened and the operating site was inspected to quantify peritoneal adhesions. Assessment of adhesions was made using the Leach score (Table 1) [29–31]. The scores for each item were added to obtain a total adhesion score between 0 and 10. For each animal, two different surgeons blinded to agent allocation performed scoring independently. If the score differed between the two surgeons, the mean score was calculated.

**Table 1:** Evaluation of adhesion using Leach score.

Extent of adhesion	Severity	Degree
0 = no adhesion	0 = no adhesion	0 = no adhesion
1 = less than 25% of the damaged area	1 = filmy and avascular adhesion	1 = adhesion is separated from tissue with a gentle traction
2 = between 25 and 50% of the damaged area	2 = vascular or opaque adhesion	2 = adhesion is separated from tissue with a moderate traction
3 = between 50 and 75% of the damaged area	3 = attachment of the cecum to the abdominal wall	3 = adhesion is separated from tissue with a sharp dissection or impossibility of separating tissues without organ damage
4 = between 75 and 100% of the damaged area		

### Evaluation of *in vivo* elimination of polymers

The macroscopic *in vivo* elimination of the agents was observed. Short-term removal for PA and PB films was observed at day 2; mid-term elimination for all products at day 5; and long-term elimination for all products at day 12. The molecular weight and kinetic of degradation of copolymer A and B was also evaluated *in vivo* by size exclusion chromatography (unpublished data).

### Histopathological evaluation

At days 5 and 12, assessment of adhesions was made using the Leach score. When there was no adhesion (Leach score = 0) the peritoneal defect was excised, and when the Leach score was positive, the adhesions between the cecum and the abdominal wall were excised. Samples were immersed in 10% formalin at room temperature for 24 hours, washed twice in Phosphate Buffered Saline (PBS) and then immersed in 70% ethanol and kept at 4°C. Then the tissue samples were embedded in paraffin. Paraffin wax blocks were cut into 3 μm thick sections. Prepared sections were then stained with hematoxylin-eosin safran (HES) and Sirius red. One histologist evaluated all tissues and was blinded to the origin of the samples.

### Statistical analysis

All quantitative values are expressed as median +/- IQ range. Statistical analysis was performed with R statistical software. The number of animals to include was estimated to demonstrate a reduction of average adhesion score of 10/10 in control group to 5/10 in the PA group. To achieve an alpha risk at 5% and power at 80% the number of animals to include was six animals in each group. Adhesion scores were globally compared using Kruskall Wallis test. The pairwise group comparisons were made by Mann-Whitney U-test. Tests of significance were two-sided with a 0.05 alpha risk.

## Results

### Animal model

**Fig 2:**
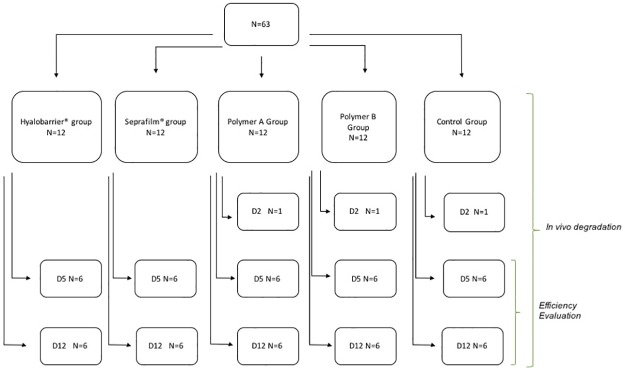
Flow chart of the protocol. Rats were randomized in five groups. In vivo degradation was evaluated at day 2, 5 and 12. Efficiency was evaluated at day 5 and Day 12.

The experimental design is shown in Fig 2. No death was observed at the time of induction of anesthesia, however one rat in PA group died at day 1 post-surgery. Autopsy showed peritoneal infection due to cecal perforation. No rat showed any sign of physical pain requiring euthanization, and no adverse events occurred.

### Evaluation of anti-adhesion efficiency

**Fig 3:**
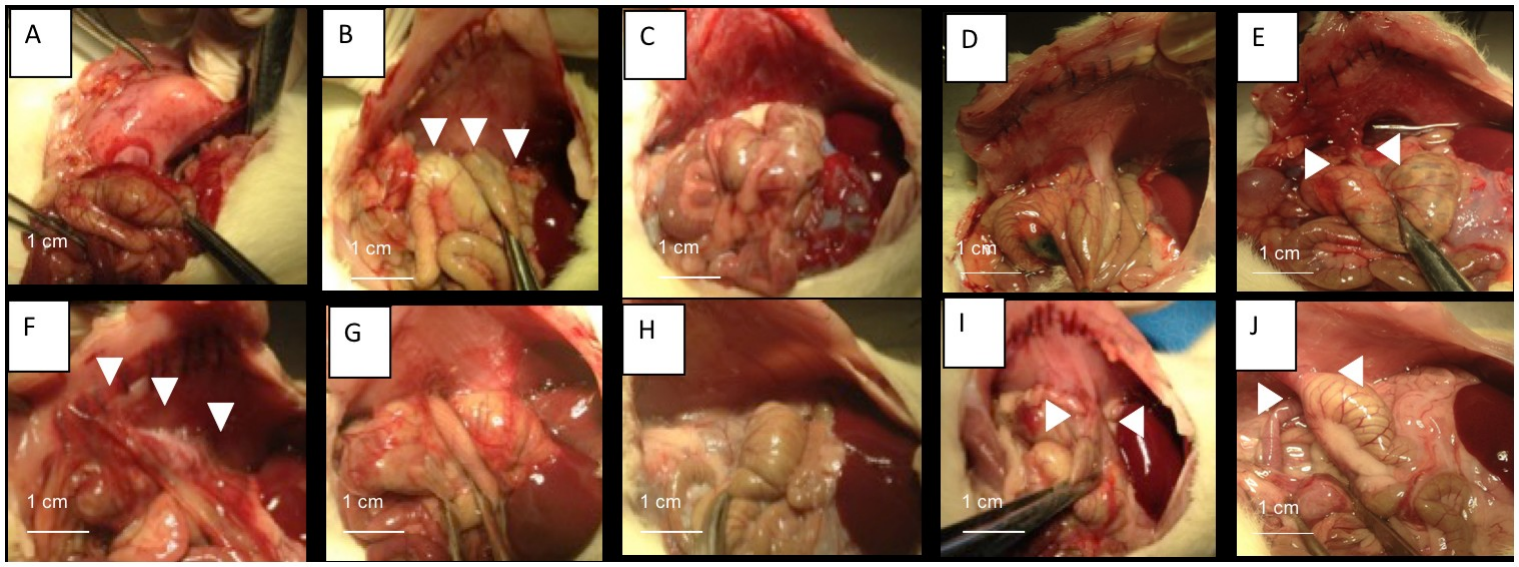
Adhesion assessment at day 5 and 12 by macroscopic examination. Necropsy pictures from different groups showing adhesions between cecum and parietal peritoneum (white arrowheads). At day 5 no adhesion was observed in Hyalobarrier group (A) or polymer A group (C), whereas strong adhesions were seen in control group (B), Seprafilm group (D) and Polymer B (E). At day 12: No adhesions were observed in polymer A group (H) whereas adhesions were apparent in Hyalobarrier group (F), Seprafilm group (I), Polymer B group (J) and control group (G).

The results of adhesion scores at day 5 and 12 are shown in Table 2. In the control group, high adhesion scores were observed (median 10 [0.25–10] at day 5, and 9 [6.75–9.75] at day 12). In Hyalobarrier group, no animal had adhesions at day 5, however, at day 12, the median adhesions score was 3.5 [0–9.25]. In Seprafilm group, the median adhesion score was 4 [0.75–6.5] at day 5 and 1.5 [0–7.5] at day 12. (Fig 3).

**Table 2:** Results of efficiency study at days 5 and 12.

	Adhesion score^a^ At Day 5 median (IQR)	p value^b^	Adhesion score^a^ At Day 12 median (IQR)	p value^b^
Group PA	0 (0–0)	-	0 (0–0)	-
Group Control	10 (0.25–10)	0.002	9 (6.75–9.75)	0.02
Group Hyalobarrier^®^	0 (0–0)	No difference	3.5 (0–9.25)	0.10
Group Seprafilm^**®**^	4 (0.75–6.5)	0.03	1.5 (0–7.5)	0.10
Group PB	2 (0–5.5)	0.07	2.5 (0–7.25)	0.25

^a^ Adhesion score calculated using Leach score

^b^ p value with Mann-Whitney test, compared to group PA

In the PA group, no animal had adhesions at either time point (Fig 3). This represents a significant difference with the control group both at day 5 and 12 (p = 0.002 and p = 0.02, respectively), and with Seprafilm group at day 5 (p = 0.03), although not at day 12 (p = 0.10). There was no significant difference in score with Hyalobarrier group at day 5 or 12 (p = 1 and p = 0.10, respectively). In the PB group, median adhesion scores were respectively 2 [0–5.5] and 2.5 [0–7.25] at day 5 and 12 with no significant difference compared to the PA group at either time point (Table 2).

### Study of *in vivo* elimination

**Fig 4:**
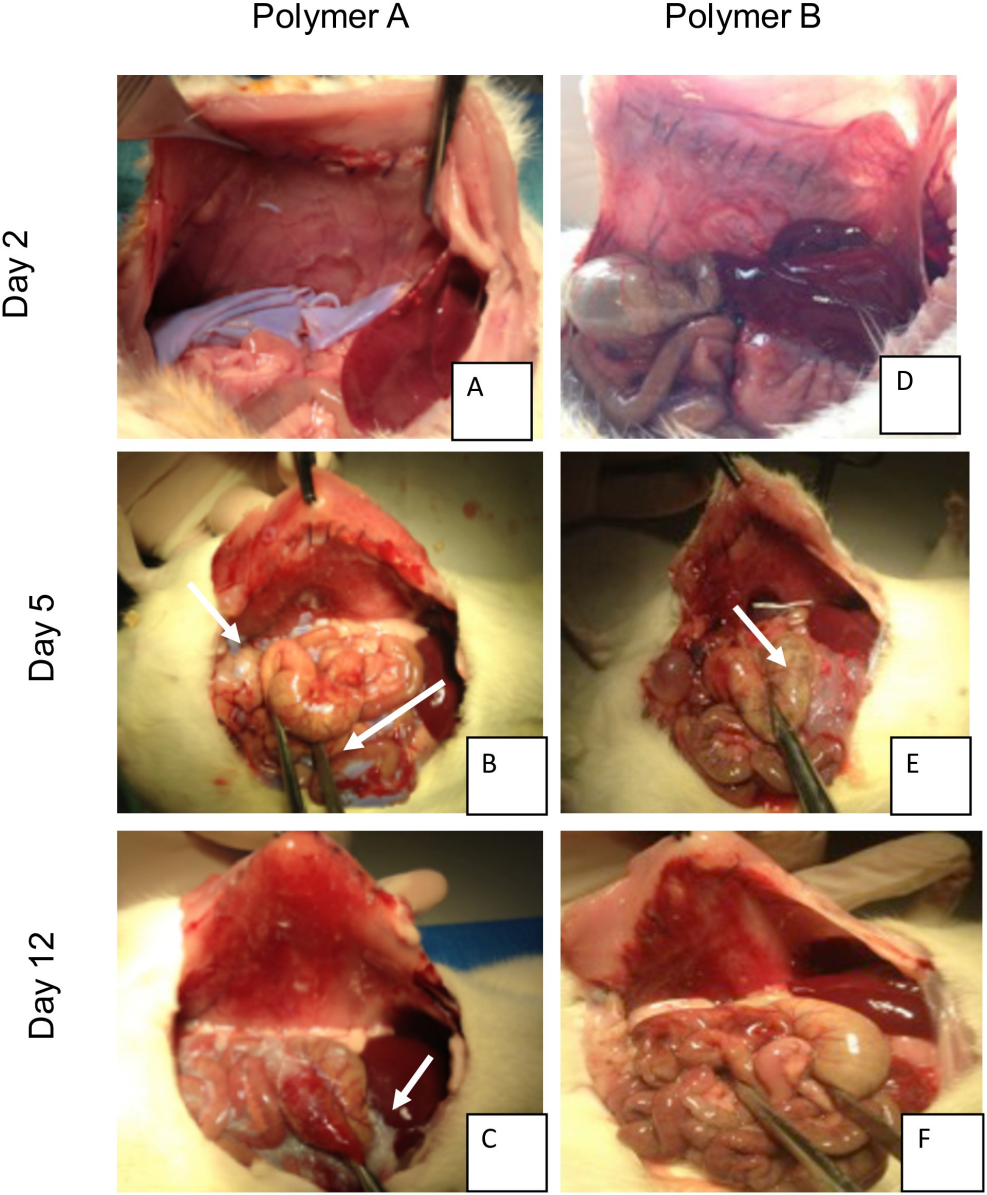
*In vivo* degradation study. Necropsy images from polymer A and B group at day 2, 5 and 12. At day 2 polymer A was intact (A), split into fragment at day 5 (white arrow) (B) and was transformed into a gel at day 12 (white arrow). Polymer B was transformed into a gel from day 2 (white arrow) (D) was in an advanced stage of degradation at day 5 (white arrow) (E) and was almost invisible at day 12 (F).

Elimination was observed at day 2, day 5 and day 12. Macroscopically, PA is almost intact at day 2, split into rough fragments at day 5 and transformed into a white gel at day 12. In contrast, PB was already transformed into a gel at day 2 and was in an advanced stage of degradation at days 5 and 12 (Fig 4).

### Histopathological examination

**Fig 5:**
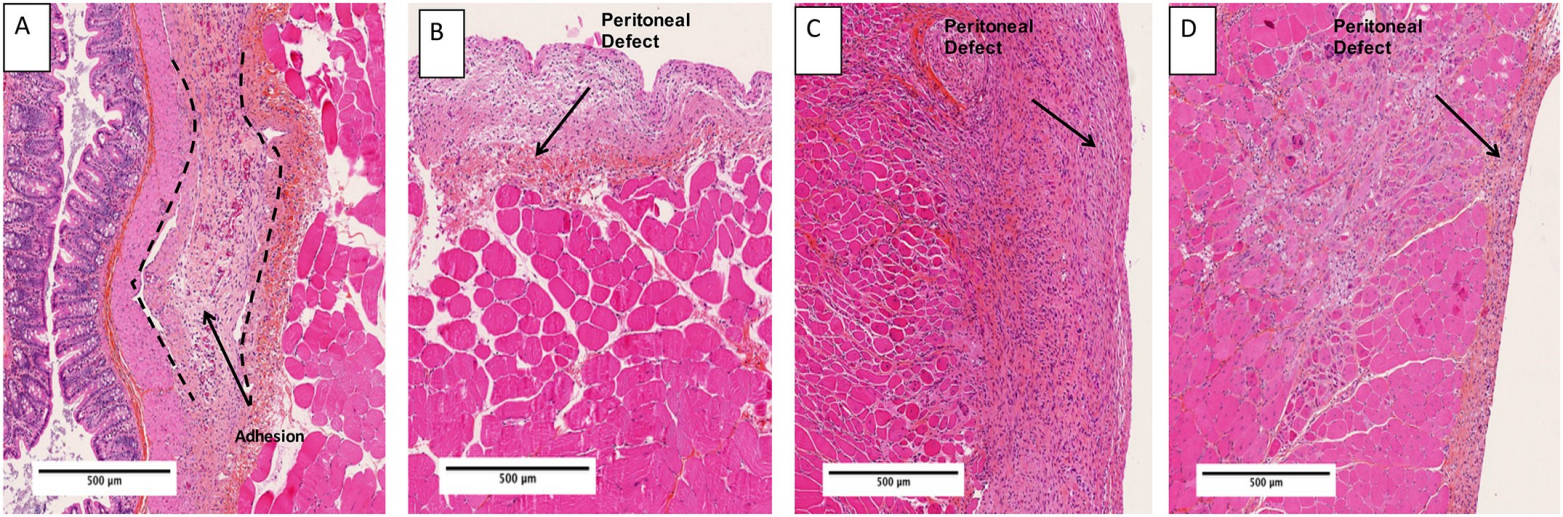
Histopathological examination at day 12. Photomicrographs from HES stained slides, magnification x5. Adhesions were visible in control group as fibrous tissue rich in fibrin, collagen and fibroblast appending the caecum and peritoneum (delineated with dashed lines) (A). Remesothalization is evident in group PA (B), Hyalobarrier (C) and Seprafilm (D) (arrows depict peritoneal defects).

In the control group, we observed adhesions between cecum and abdominal wall at day 5 and 12. There was fibrous tissue rich in fibrin, collagen and fibroblast appending the cecum and peritoneum. At day 12, fibrous tissue was more organized and neovascularization was evident (Fig 5).

In PA and PB groups, the peritoneum commenced healing from day 5 with reepithelialization on all surfaces of the abdominal wall. At day 5, inflammatory cells were prominent beneath the mesothelium layer and tissue was rich in collagen. At day 12, inflammatory cells decreased, and scar peritoneum was rich in fibrin and collagen compared to normal peritoneum.

In the Hyalobarrier group and for samples with Leach score 0, peritoneum is healing from day 5. There was a layer of inflammatory cells, and fibroblasts and collagen fibers were observed below the mesothelium layer. Inflammatory cells were still present at day 12 and the fibrin layer was thicker than in PA and PB group.

In the Seprafilm group, adhesions were visible on cecum and peritoneum samples. In the peritoneum sample, all defects were reepithelialized. As with the PA and PB groups, inflammatory cells were present at day 5 and decreased at day 12 (Fig 5).

Histological analysis demonstrates that PA did not delay the healing process. In both polymer groups, there was no excessive inflammatory response and fibrotic response was lower than in the Hyalobarrier group.

## Discussion

Barrier adjuvants are used to prevent adhesions by decreasing the apposition of injured visceral and parietal peritoneum until remesothelialization occurs [29]. The ideal barrier should not only be anti-adhesive, biocompatible, absorbable, and able to be applied through the laparoscope, but also should remain in place on the traumatized surfaces without sutures or staples, including bleeding surfaces, and not interfere with remesothelialization [32]. For many years, polymers have been used in the conception of adjuvant barriers including solid membranes, hydrogels or solutions [33].

In this study, we showed an anti-adhesion efficiency in prevention of peritoneal adhesions compared to a control and compared to two existing products on the market. The new polymer PA seems more efficient than Seprafilm at day 5 but this superiority is not significant at day 12. In comparison to Hyalobarrier the polymer PA seems to be equivalent. A supplementary study with more animals could help identify any difference. However, at this time we have not sufficient evidence to conclude that our polymer is superior to the currently available barrier.

Seprafilm consists of hyaluronic acid, which has anti-adhesion properties, and carboxymethylcellulose; it has been shown to be efficient in seven randomized controlled trials [34–40]. According to the Cochrane review into anti-adhesion prophylactic agents, this is the only agent which has an anti-adhesion efficiency in abdominal surgery [41]. It is transformed into a gel within 24 hours [42] and definitively eliminated from the organism in 28 days [43]. However, for malleability and ease of use, the galenic form of our polymer seems better suited than Seprafilm, which is very difficult to employ, in particular in laparoscopy [33,43]. Indeed, the PLA-PEG-PLA triblock has been used in healtcare for tissue engineering and seems to have good mechanical properties [44,45].

The advantage of the new device is its malleability; it does not adhere to gloves, and has to be in contact with tissue or liquid to be adhesive. If initially incorrectly placed, it could be easily repositioned, contrary to Seprafilm. Furthermore the galenic form in film could allow it use and residence inside the uterus by hysteroscopy contrary to a gel.

PEG is known for its anti-adhesion properties and is incorporated in products already on the market. Two randomized clinical trials [20,46] showed a significant decrease of adhesions after the use of PEG in myomectomy surgeries. Ten Broek et al. conducted a meta-analysis including 85 articles on the efficiency of PEG in gynecological surgery showing a significant decrease of the incidence by 0.27 (IC 95% 0.11–0.67) when PEG was used [46].

Time of residence of PA appears to be compatible with the peritoneal adhesion formation. Our results suggest that adhesion formation starts between the second and fifth postoperative day, in agreement with the literature [6,33]. Compared to PA, the elimination of PB seems too fast for prevention of peritoneal adhesions.

Histologic analysis demonstrated the safety of PA; it is biocompatible, does not interfere with the healing process and does not induce specific inflammatory response.

The main limitation of this study was the use of an animal model, which cannot substitute a human model, although several studies have validated this animal model and this model was used to test many devices currently on the market for human use [29–33]. However, bleeding is responsible for an increase of adhesion [7,47], a feature not present in this model; as a consequence, the effect of these materials on the abdominal wall of the animals does not necessarily reflect their properties in the human body in routine gynecology surgery. Future clinical studies are needed to confirm our results. The evaluation in a rat model may not reflect their properties in another animal models. But some PEG copolymer has been evaluated in other animal models as rabbit and also reported a decrease of adhesion with the use of PEG polymer [19,25]. Other barriers agents have been tested in the same cecal abrasion model but with mice and are in the line with our findings [48]. This is in favor of a homogeneity of results if our polymer will be used in other models. Our study shows heterogeneous results for adhesions score, probably because two different surgeons evaluated the adhesions, however our results remained significant compared to the control group. This study was also limited by the comparison with only two anti-adhesive agents in parallel. We chose them as they represent the anti-adhesive agents most frequently used in gynecological surgery. The short duration of follow up could also be a limitation; however abdominal adhesions form within seven days following surgery, thus we considered that the evaluation at day 12 was representative of the long-term evaluation [7], particularly as OFA rats have faster healing abilities than humans.

We observed one death in our study in the PA group only. This death occurred before euthanasia, one day after surgery. Autopsy showed peritoneal infection due to cecal perforation. This perforation probably occurred during the initial surgery with cecal abrasion because it happened very prematurely. As such, it is unlikely that this death was linked to the device, but we cannot exclude that possibility. Furthers studies are required to develop this product, especially the evaluation in contaminated environment [49] or on the progression of cells tumor.

## Conclusion

In conclusion, results of this study suggest that the PLA-PEG-PLA films were efficient at limiting the incidence of peritoneal adhesions in an experimental model of adhesions. In our study, the performance of this new device was comparable with those currently used in clinical practice. Moreover, its ease of use and time of residence represents a real advantage over the other devices. Our polymer film could become an essential tool for the surgeon if its efficiency is confirmed in clinical trials. It seems to be useful and safe for clinical practice and to prevent the adhesion formation after abdominal surgery.
